# Advances in the Research and Development of Breast Cancer Organoids

**DOI:** 10.32604/or.2026.083636

**Published:** 2026-08-13

**Authors:** Ling Li, Shuai Zhao, Xiaoxiao Wang, Jiahui Du, Zhen Jin, Song-Bai Liu, Xiaohua Li

**Affiliations:** 1 School of Chemistry and Life Science, Biology and Materials Engineering, Suzhou University of Science and Technology, Suzhou, China; 2 Jiangsu Province Engineering Research Center of Development and Translation of Key Technologies for Chronic Disease Prevention and Control, Suzhou Vocational Health College, Suzhou, China; 3 Thyroid and Breast Surgery, Wuzhong People’s Hospital of Suzhou City, Suzhou, China

**Keywords:** Breast cancer organoids, tumor microenvironment, drug screening, personalized medicine

## Abstract

Breast cancer ranks first in global cancer incidence. Due to its high heterogeneity, cancer cells often develop drug resistance during metastasis, leading to therapeutic challenges and poor prognosis. Consequently, breast cancer organoid models have emerged, which can effectively recapitulate the tumor microenvironment and serve as important tools for investigating the mechanisms underlying breast cancer initiation and progression. Breast cancer organoids exhibit interactions between cells and the extracellular matrix (ECM) while retaining the heterogeneity of the original tumor cells. Owing to these advantages, such models have been widely applied in studies of tumor pathogenesis, disease modeling, drug screening, therapeutic response prediction, and microenvironment reconstruction. Although breast cancer organoids still have certain limitations in terms of construction, long-term culture, and fully recapitulating the tumor microenvironment, they represent an emerging technology that promotes the development of high-throughput drug screening and precision personalized therapy. At the same time, the integrated culture of breast cancer organoid models with immune cells or endothelial cells and microfluidic chips was also explored. This approach enhances the complexity of the *in vitro* tumor microenvironment, making it closer to the human microenvironment, thereby improving the authenticity and reliability of preclinical research data. Compared with traditionally statically cultured breast cancer organoids, it possesses more dynamic regulatory characteristics and is more accurate in simulating the tumor microenvironment. The aim of the study is to discuss the applications of breast cancer organoids and to incorporate new technologies into traditional breast cancer organoid models, making the research outcomes more clinically relevant.

## 1 Introduction

Breast cancer is the most frequently diagnosed malignant tumor among women worldwide [1]. Among 185 countries, it ranks as the most common cancer in women in 157 countries and is the leading cause of cancer-related death among women in 112 countries. According to the World Health Organization (WHO), 666,000 people died from breast cancer, making it the fourth leading cause of cancer death globally and accounting for 6.9% of all cancer deaths. WHO estimated that in 2022 there were 2.3 million new cases of breast cancer worldwide, accounting for 11.6% of all cancers, with 670,000 deaths. It is projected that by 2050, the number of breast cancer cases will increase to 3.6 million, and the number of deaths will reach 1.1 million. Although most people generally consider breast cancer to be a disease affecting women, the WHO reports that approximately 0.5%–1% of cases occur in men.

Breast cancer is a heterogeneous disease, and different subtypes show significant differences in genetic characteristics, histological morphology, and clinical treatment. Among clinically diagnosed breast cancers, about 70% are estrogen receptor (ER)-positive [2]. From the perspective of the above molecular classification, approximately 60% of breast cancers are luminal-type breast cancer, while HER2-positive breast cancer accounts for about 20% [3]. The abnormal proliferation of breast cancer is mainly driven by multiple factors, including genetic susceptibility, environmental exposure, and lifestyle [4]. The rapid proliferation of breast cancer leads to drug resistance of cancer cells and cancer metastasis, thereby making treatment difficult and resulting in poor clinical prognosis [5].

The formation of breast cancer is a complex multi-step process, but the exact mechanism of its carcinogenic progression remains unclear. For patients who have already been diagnosed with breast cancer, clinical treatments commonly include radiotherapy, hormone therapy, surgery, and chemotherapy. Drug resistance of tumor cells, recurrence of cancer cells, and distant metastasis are the main causes of breast cancer-related death. Therefore, there is an urgent need to develop an *in vitro* model that can accurately simulate the unique molecular subtypes of breast cancer in patients, in order to enable personalized treatment for breast cancer patients. Breast cancer organoid models can simulate the patient-specific tumor microenvironment *in vitro* and provide an effective platform for breast cancer research.

Stem cells from human tissues are cultured in a three-dimensional (3D) culture matrix. Under specific spatial and lineage constraints, organ-specific cells form structures that mimic native organ architecture through adhesion, aggregation, self-organization, and differentiation [6]. It is a 3D cellular structure that can exhibit cellular architecture, cellular heterogeneity, and tissue functions *in vitro* [7]. This recapitulates the structure and function of human organs, simulates stable genomic transcriptional and epigenetic profiles, and can be maintained in long-term, stable culture *in vitro*. In cancer research, organoid models derived from induced pluripotent stem cells (iPSCs) and adult stem cells preserve the integrity of the genome in tumor tissues and are more often used to study disease mechanisms and biological processes [8]. In contrast, organoids directly obtained from patients’ tumor tissues are more often used to recapitulate the tumor microenvironment and provide a basis for precision medicine and drug use [9].

Breast cancer organoids are like a translational platform, which can simulate and present the complexity of *in vivo* physiology for *in vitro* study [10]. Cells growing in the complex *in vivo* microenvironment are regulated by multiple signals and interactions, thereby establishing and maintaining specific phenotypes and functions. With the rapid development of 3D culture technology, organoid models are able to simulate this complex microenvironment [11]. Organoids exhibit consistency with the original cells and are able to replicate and simulate the specific biological functions of the parent cells [12]. Therefore, organoids are used to study the characteristics of interactions between cells as well as between cells and the matrix [13].

Tumor tissues are enzymatically digested and dissociated after surgical resection or lesion biopsy, and then cultured to generate patient-derived organoids (PDOs) [14]. PDOs can recapitulate the phenotypic and genetic characteristics of the original tissue *in vitro*. Organoid models established from surgical or punch biopsy samples can maintain genomic stability while also reproducing the heterogeneity and diversity of the original tumor [15,16]. PDOs transform traditional cancer cell line culture methods into a 3D, patient-specific *in vitro* differentiation system of cancer cells. PDOs simulates the structural composition, biological functions, and physical properties of the cellular basement membrane in the human body. PDOs also includes tumor cell heterogeneity, gene and protein expression, and certain metabolic activities, making it more consistent with the physiological microenvironment of the human body [17]. Using PDO models, the heterogeneity and molecular characteristics of the original tumors can be well preserved, facilitating more accurate studies of tumor growth, drug responses, and mechanisms of drug resistance.

Early detection of breast cancer is difficult, cancer cells proliferate rapidly, the likelihood of recurrence is high, and metastasis to other sites occurs easily, resulting in a lack of effective targeted therapies. Breast cancer organoids are complex multicellular 3D models that enable effective investigation of the proliferation process of breast cancer [18]. They are therefore crucial for the precise study and treatment of breast cancer, as well as for identifying new diagnostic and prognostic biomarkers and developing advanced therapeutic strategies [19]. Breast cancer organoid models are not only widely applied in drug screening and molecular medicine, but their value in basic biological research is also increasingly evident. The aim of the study is to discuss the applications of breast cancer organoids and to incorporate new technologies into traditional breast cancer organoid models, making the research outcomes more clinically relevant.

## 2 Research on Breast Cancer Organoids

### 2.1 Establishment of Breast Cancer Organoids

#### 2.1.1 Early Development of Three-Dimensional Breast Cancer Cultures and Advances in In Vitro 3D Culture Techniques

As early as 1979, Mary Jane Emerman and colleagues first attempted to culture normal mammary epithelial cells in collagen gels [20]. The collagen gel provided a unique microenvironment that allowed cells greater access to nutrients, thereby promoting the growth and structural differentiation of mammary epithelial cells [21]. In 1982, Mina J. Bissell et al. first applied organoid culture techniques in the study of breast cancer tumors and found that the ECM regulates gene expression in breast cancer cells [22]. These findings demonstrated the importance of the microenvironment in tumor research and shifted breast cancer research methods from two-dimensional models to 3D models.

Systematic studies of the microenvironment in breast cancer cells have laid the foundation for the successful establishment of breast cancer organoid models. In 1982, M. Hiratsuka et al. successfully isolated and cultured a breast cancer 3D culture with ductal structures from breast biopsy tissues with minimal fibroblast contamination [23]. In 1992, Petersen first referred to 3D tissue structures as “organoids”. In the same year, Mina J. Bissell developed a method to culture normal primary mammary epithelial cells and biopsy-derived cancer cells using basement membrane, laying the foundation for identifying tumor suppressor genes [24]. In 2007, Mina J. Bissell et al. compared two 3D culture methods for breast cancer cells [25]. The cells were either embedded in or seeded onto a laminin-rich extracellular matrix (lrECM). Their study found that an embedded 3D culture of breast cancer cells had a longer growth cycle than culture on top of the matrix. Using lrECM allowed the detection of cell-cell interactions, and compared with traditional two-dimensional cell culture, it enabled the observation of signaling pathways within the microenvironment [26]. In 2013, the first patient-derived breast cancer organoids were established by Drose and colleagues [27]. Researchers obtained breast cancer samples through surgical tumor resection and added collagenase and hyaluronidase to the fragmented tumor tissue to isolate breast cancer cells through enzymatic digestion. In this study, they also found that differences in the source of breast tumor samples required different digestion and culture conditions for organoid establishment. In 2015, Zubeldia-Plazaola et al. used organoid technology to culture primary mammary epithelial cells [28]. Their team found that improving the success rate of breast cancer organoid culture requires slow digestion at low enzyme concentrations. This study provided an effective approach for further analyzing the functional differences among epithelial cell types involved in the origin of breast cancer.

#### 2.1.2 Advances in the Applications of Breast Cancer Organoid Models

**Figure 1:**
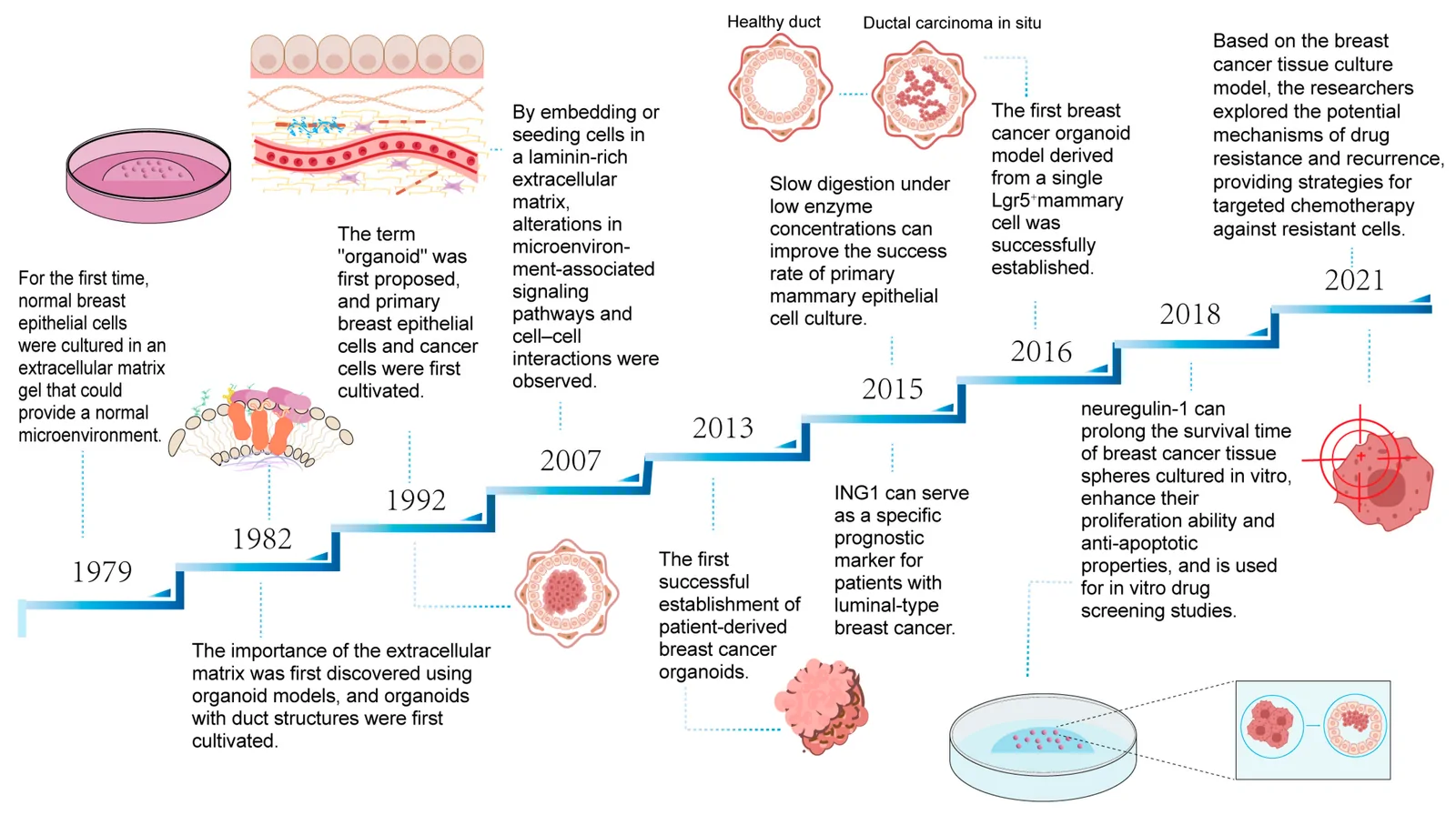
**Development history and research timeline of breast cancer organoids.** In 1979, normal mammary epithelial cells were first cultured. In 1982, organoid technology was first applied to breast cancer cells, revealing the importance of the tumor microenvironment and enabling the culture of breast cancer organoids with ductal structures. In 1992, the term “organoid” was first introduced, and the basement membrane was used to culture mammary cells and cancer cells. In 2007, two 3D culture methods were used to culture breast cancer organoids, demonstrating the signaling role of the tumor microenvironment. In 2013, patient-derived breast cancer organoids were successfully established for the first time. In 2015, the culture method for breast cancer organoids was improved, allowing breast cancer samples to be digested and cultured under low enzyme concentrations. In 2016, a breast cancer organoid model based on single Lgr5+ mammary cells was constructed for the first time. In 2018, it was discovered that neuregulin-1 can prolong the survival time, proliferative capacity, and anti-apoptotic properties of breast cancer organoids *in vitro*, enabling efficient establishment and long-term maintenance. In 2021, breast cancer organoid models were used to study the mechanisms of drug resistance and recurrence in breast cancer, providing new therapeutic strategies for targeting chemotherapy-resistant cells. (Using Adobe Illustrator 2024 to create images).

The successful establishment of breast cancer organoid models has promoted their widespread application across multiple fields. Satbir et al. investigated the expression levels of cytokines and matrix metalloproteinases (MMPs) in breast fibroblasts overexpressing inhibitor of growth family member 1 (ING1) [29]. They co-cultured stromal cells with organoids derived from MCF-7 cells and found that ING1 increased MMP expression while downregulating tissue inhibitors of metalloproteinases (TIMP), thereby promoting the progression of breast cancer cells. Based on the specificity of ING1, luminal breast cancer patients were evaluated to provide support for improving patient prognosis. In 2016, Zhang et al. added exogenous factors such as epidermal growth factor (EGF), Wnt-3A, and R-spondin to the organoid culture medium [30]. Mouse Lgr5+ mammary cells were cultured in a medium supplemented with exogenous factors that activate the Wnt signaling pathway. Under these conditions, a breast cancer organoid model based on single Lgr5+ mammary cells was constructed for the first time. This study found that endogenous Wnt signaling in stem cells mediates the growth and proliferation of breast cancer organoids. It can serve as a potential model for studying hormone-regulated ductal development. This type of organoid model enables in-depth analysis of regulatory interactions in breast cancer cells from the perspective of signal transduction. In 2018, Norman Sachs et al. successfully established 95 breast cancer organoids from 155 breast cancer samples [31]. Using next-generation sequencing (NGS) data analysis, they found that organoids can retain copy number variations (CNVs), single-nucleotide variants (SNVs), and neuregulin-1. Neuregulin-1 is a ligand for human EGF receptor tyrosine kinases 3 and 4, which can prolong the survival time, proliferative capacity, and anti-apoptotic properties of breast cancer organoids *in vitro*. Efficient establishment and long-term maintenance of breast cancer organoids require the support of neuregulin-1. The successfully established models were used for *in vitro* drug screening studies. In 2021, Eugen et al. used patient-derived breast cancer organoid models to study the molecular characteristics of residual breast cancer cells after clinical treatment [32]. They explored the mechanistic links between different tumors and molecular environments in regulating cellular stress sensing and dormancy-like adaptation. Persisting tumor cells can reduce drug cytotoxicity through molecular adaptation mechanisms similar to embryonic diapause, including depletion of the oncogene Myc or inhibition of its transcriptional coactivator bromodomain-containing protein 4 (Brd4). Treatment-persistent organoids based on breast cancer organoids were used to investigate the mechanisms of drug resistance and recurrence, thereby providing new potential strategies for targeting chemotherapy-resistant cells. The development and research progress of breast cancer organoids are shown in Fig. 1.

Breast organoids provide an important model for studying key factors that influence signaling pathway regulation, changes in gene expression, and tissue structure remodeling. They are an important model for *in vitro* biological research and play a crucial role in simulating physiological processes of tumor tissues, disease modeling, and drug screening [33].

### 2.2 Advantages of Breast Cancer Organoids

Breast cancer organoid models involve the 3D culture of breast tumor tissues derived from patients. Organoid models simulate the key morphological and functional characteristics of breast tumor tissues, thereby enabling more effective studies of tumor biology. Breast cancer organoids can be maintained in long-term culture to model the unique genetic complexity and phenotypic heterogeneity of breast cancer [34].

#### 2.2.1 The Importance of the Microenvironment in Breast Cancer Organoids

Throughout the initiation and progression of breast tumors, the microenvironment plays a crucial role. The tumor microenvironment of breast cancer includes fibroblasts, mesenchymal stem cells, macrophages, adipocytes, and the ECM. ECM is composed of laminin, type IV collagen, fibronectin, and heparan sulfate proteoglycans [35]. These microenvironmental components act synergistically in promoting cancer cell growth, invasion, and metastasis, with interactions between cancer cells and the ECM serving as a hallmark for studying the tumor microenvironment [36]. ECM proteins can be stiffened under the influence of cancer cells, which increases the invasiveness of cancer cells while reducing patient survival rates [37]. Therefore, the study of the ECM is indispensable in breast cancer organoid research. The breast cancer tumor microenvironment exhibits a unique cellular composition and significant spatial heterogeneity. It typically presents with low extracellular pH and a highly hypoxic state, conditions that promote the growth and survival of breast cancer cells [38]. Cancer cells recruit other cells into the tumor region by secreting various growth factors and chemokines [39]. In breast cancer organoid models, it is essential to simulate the complex tumor microenvironment to study cell-cell interactions. A limitation of miniature tumors cultured in dishes is the lack of immune cells and a complete vascular network, which are key components of the microenvironment and are also lacking in traditional breast cancer organoid models.

#### 2.2.2 Advantages Compared with Other Models

Two-dimensional cell culture models lack the simulation of interactions between cells and the ECM and cannot preserve patient-derived 3D tissues [40]. This often leads to genetic drift and loss of heterogeneity, resulting in inaccurate experimental outcomes [41]. In contrast, breast cancer cells interact with each other as well as with surrounding non-malignant cells, hormones, secreted factors, and the ECM [42]. For example, Jennifer M. Rosenbluth et al. found that immortalized HMECs obtained from 2D culture can form regular cyst-like or squamous-centered structures in a 3D culture system [43]. However, the culture conditions make it difficult to maintain ER+ luminal cells over the long term, and their biological characteristics differ significantly from those of primary mammary epithelial cells. Breast cancer organoid models better reflect the predicted responses after tumor treatment. Moreover, breast cancer organoids are 3D models that allow cells to grow in all directions, and this growth pattern more accurately simulates interactions between cells and the ECM [44].

Compared with organotypic tissue slices, breast cancer organoids can be preserved for a longer period and better reflect the stem cell potential and specificity of tumor tissues. These models retain the heterogeneity of breast cancer cells. Organoid models constructed from patient breast tumor samples obtained via biopsy or surgery provide relatively personalized and precise treatment for patients. Breast cancer organoid models preserve the genetic characteristics of tumor tissues as well as the tumor microenvironment. The advantages of organoid models allow breast cancer to be analyzed *in vitro*, thereby enhancing clinical research on breast cancer. Breast cancer organoids are not only used for high-throughput drug screening but also serve as key models for validating drug efficacy [45]. By recapitulating the characteristics of patient tumor tissues, they provide strong support for experimental evaluation of drug responses and for the development of more direct and precise personalized treatment strategies in clinical practice.

Patient-derived xenograft (PDX) models can maintain tumor heterogeneity. However, they are associated with several limitations, including low engraftment rates, long timeframes for model establishment and drug screening, high cost, lack of immune cells, and significant differences in drug metabolism due to species differences between humans and mice. They are not suitable for high-throughput screening, and they are typically established from late-stage tumors [46]. Breast cancer organoids can better recapitulate human biological characteristics and are more aligned with clinical needs in translational applications. Breast cancer organoids can be generated from small biopsy samples and can accurately predict patient drug responses after treatment; however, they require relatively high culture costs.

A certain proportion of breast cancer patients still experience recurrence after being cured, and current approaches in personalized oncology struggle to consistently identify effective biomarkers of treatment response [47]. Therefore, breast cancer organoid models are also of great significance in evaluating the risk of recurrence in high-risk early cancer cells.

### 2.3 Application of Breast Cancer Organoids

#### 2.3.1 Research on the Pathogenesis of Breast Cancer Organoids

**Figure 2:**
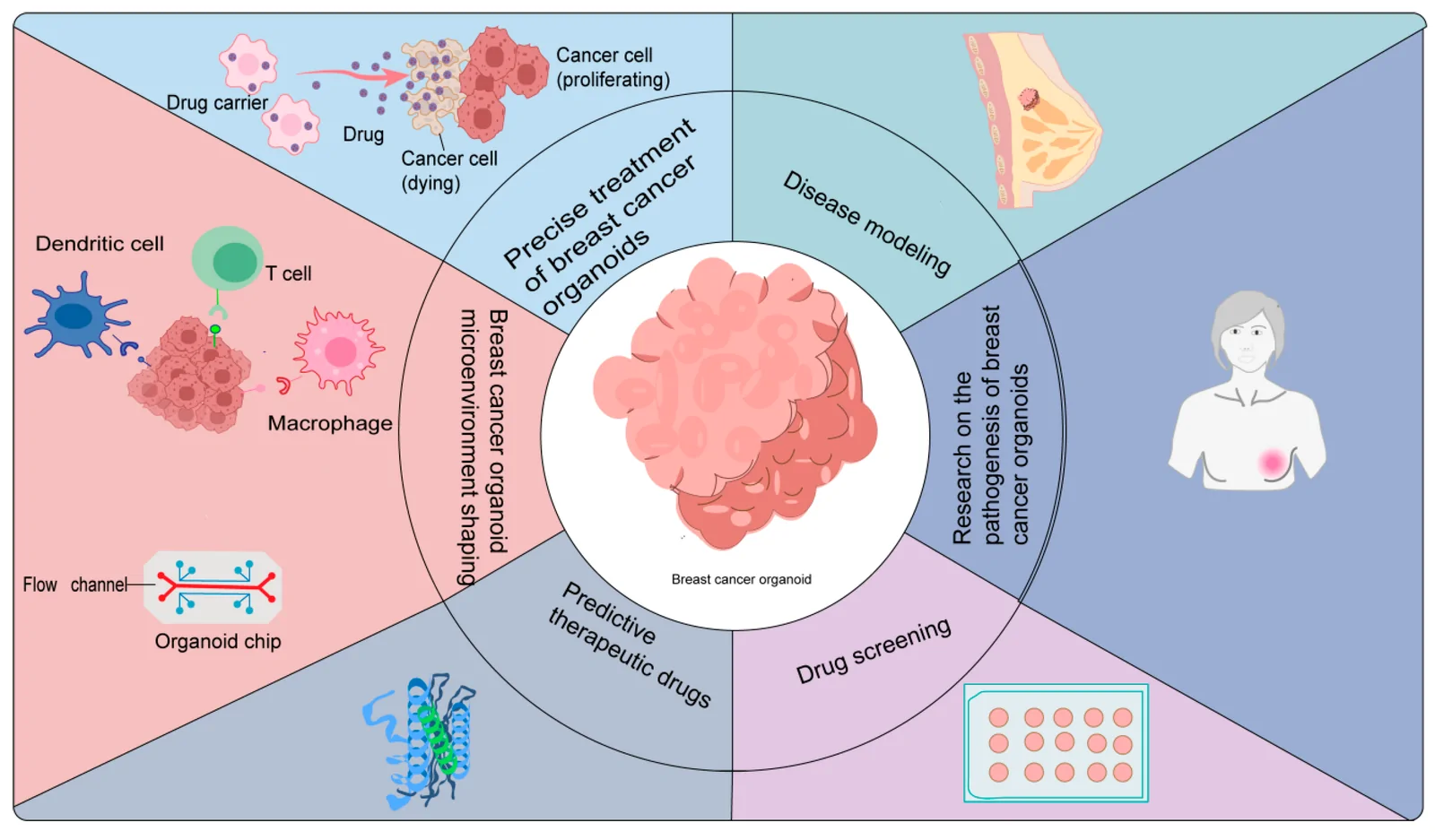
**The widespread applications of breast cancer organoids.** Breast cancer organoids are mainly applied in tumor pathogenesis research, disease modeling, drug screening, therapeutic drug prediction, and tumor microenvironment engineering, demonstrating broad versatility across these areas. (Using Adobe Illustrator 2024 to create images).

Biobanks of organoids derived from normal patient mammary tissue and breast cancer tissue have been established. These organoid models recapitulate genomic alterations, transcriptomic signatures, specific cell types, and morphological characteristics of both breast tissue and breast cancer cells, thereby enabling better investigation of novel targets related to tumor heterogeneity during the initiation and growth of tumor lesions [48]. This provides a convenient and scientifically robust model for clinical breast cancer treatment (Fig. 2). Previous studies have explored the application of breast cancer organoids in investigating the mechanisms of bilateral breast cancer. It was found that, although different lesions may present distinct molecular subtypes, the early core mutations are identical, and organoids are consistent with primary tumors in both driver gene profiles and clonal architecture. Breast cancer organoids can therefore serve as an *in vitro* model to trace the common origin and early oncogenic events of breast cancer [49]. The view that breast cancer organoids can faithfully recapitulate patient tumor characteristics was confirmed by Mamta Kumari in 2025 [50]. Using breast cancer organoid models to simulate the initiation and progression of breast cancer, we reveal that the organoids are consistent with the patient’s primary tumors in terms of morphology, molecular subtypes, and key driver mutation profiles.

Breast cancer organoid models, combined with multi-omics analyses and functional experiments, have been used to investigate the key molecular pathways involved in circulating tumor cell metastasis and the development of drug resistance [51]. The tumor suppressor gene TP53 is one of the most frequently mutated genes in human cancers and plays an important role in regulating cell cycle arrest, DNA repair, apoptosis, and senescence responses. In gene editing and functional studies, breast cancer organoids have been used to investigate key driver genes such as TP53, PIK3CA, RCAI, and HER2, as well as to explore the mechanisms by which abnormal signaling pathways contribute to tumor cell initiation, progression, and differentiation [52]. It has been found that organoids can retain different tumor subpopulations, allowing the study of breast cancer heterogeneity, progression, and the molecular basis of therapeutic stress responses, thereby revealing mechanisms underlying invasion, metastasis, and drug resistance. When investigating the effects of driver mutations on cell signaling, tumor heterogeneity, and progression, breast cancer organoid models are commonly used. They help elucidate key cellular behaviors and structural bases underlying tumor invasion and metastasis, including collective migration, cell adhesion, epithelial-mesenchymal transition, and the tumor-suppressive role of myoepithelial cells as a physical barrier. By constructing breast cancer organoids containing extracellular matrix components, hormonal signals, and multiple stromal cell types. Researchers can recapitulate the tumor microenvironment *in vitro* and study the regulatory roles of stromal remodeling, mechanical property changes, and immune factors in breast cancer initiation and progression [53].

Breast cancer organoids are an effective theoretical model for studying the mechanisms and evolutionary patterns of breast cancer. They can reconstruct tumor cell plasticity and metastatic potential *in vitro* while stably preserving the patient-specific characteristics of tumors over the long term. Organoid models provide a unified and controllable human-derived disease model for investigating the functions of key mutations, the mechanisms underlying tumor heterogeneity, and individualized therapeutic responses. Moreover, breast cancer organoids can simulate the initiation and progression of breast cancer at multiple levels, including genetic features, tissue architecture, and the microenvironment, making them an important experimental model for studying the pathogenesis of breast cancer.

#### 2.3.2 Disease Modeling

Human disease modeling is based on genetic engineering and genetics, and animal models were used prior to the establishment of organoid models. Animal models have ethical concerns and physiological differences from humans. The development of organoid models has enabled more in-depth study of diseases, and organoids can be directly derived from tumor tissues, allowing for more efficient translation of biological research (Fig. 2) [54].

Maja Starostecka et al. used breast cancer organoid models to systematically analyze doxycycline-induced genomic alterations at the single-cell level [55]. Using Strand-seq sequencing, they found that doxycycline treatment significantly increased the frequency of structural variations (SVs) and aneuploid chromosomes in organoids. Drug induction caused extensive DNA damage and activation of homologous recombination repair in organoids. The genomic instability induced by doxycycline in organoids is crucial for studying tumor heterogeneity and drug resistance. Breast cancer organoid models can be used to elucidate the precise mechanisms of chemotherapy-induced DNA damage. Based on these mechanisms, different therapeutic strategies can be developed to improve cancer treatment and reduce the probability of tumor recurrence.

A key factor in cancer treatment is the complex crosstalk between breast cancer cells and their neighboring stromal cells. Breast cancer organoid models are 3D culture systems containing matrix gel, which can simulate cellular responses within the tumor microenvironment, as well as the complex structure and functions of the original tumor. Studies using breast cancer organoid models have shown that these models can effectively preserve the heterogeneity and crosstalk characteristics of epidermal growth factor receptor-positive (EGFR+) tumor cells and platelet-derived growth factor receptor β-positive (PDGFRβ+) stromal cells, and can be used to investigate the synergistic effects of photodynamic therapy (PDT) and photothermal therapy (PTT) [56]. As preclinical disease models, breast cancer organoids can not only be used to study the tumor-specific cytotoxic effects of dual-targeting nanoplatforms in patients, but also provide direct experimental data to support the clinical application of nanotherapeutic strategies.

Due to the significant limitations of traditional two-dimensional cell culture in simulating the dynamic evolution of breast cancer tumors, researchers have gradually shifted to breast cancer organoid models to mimic the processes of tumor initiation and progression. Johanna F. Dekkers et al. used CRISPR/Cas9 gene-editing technology to introduce breast cancer-related mutations into normal human mammary organoids, successfully constructing an *in vitro* model of breast cancer development [57]. This model systematically recapitulated the transformation from normal epithelial cells to malignant tumors, providing a controllable human-derived disease model for studying the origin, molecular subtypes, and driving mechanisms of breast cancer. In 2018, Sabra L. Djomehri et al. established a reproducible mammary organoid model capable of stably simulating normal mammary-like structures and the progression of breast cancer [58]. Through TGF-β stimulation, hypoxia induction, and co-culture with stromal cells, the organoids exhibited tumor progression features such as epithelial-mesenchymal transition, lumen loss, and increased invasiveness, while also retaining the morphological characteristics of primary tumors. This demonstrated that the system can serve as an effective disease model for studying the mechanisms of breast cancer development and the role of the microenvironment. In addition, by constructing patient-derived breast cancer organoids that recapitulate the biological characteristics of primary tumors at the levels of tissue architecture, molecular features, and function, researchers have successfully simulated tumor evolution before and after neoadjuvant therapy [59].

Breast cancer organoid models can not only reflect the mechanisms of breast cancer initiation and progression and simulate the tumor microenvironment, but also be used to study treatment-induced changes in tumor heterogeneity and malignant progression, thereby revealing tumor evolutionary patterns and advancing precision oncology research. They represent a reliable *in vitro* platform for breast cancer disease modeling.

#### 2.3.3 Drug Screening

Organoids are rapidly becoming the gold standard for drug screening. Due to the high heterogeneity of breast cancer tumors, clinical responses to individualized treatments vary significantly. Therefore, there is an urgent need to develop models that can monitor tumor progression in patients for more precise drug research. The use of breast cancer organoid models for drug screening has been proven to be an effective tool for the treatment of breast cancer (Fig. 2).

Using breast cancer organoid models, an *in vitro* screening of 49 clinically relevant drugs was performed [60]. Experiments confirmed that different breast cancer organoids exhibit significant heterogeneity in response to chemotherapeutic agents and targeted therapies, and can identify potential therapeutic sensitivity targets in different patients. Researchers conducted assays on apoptotic markers, cell cycle proteins, and signaling pathway activity in organoid models to validate the molecular mechanisms of drug action. Based on this, combination therapy regimens were evaluated, and potential synergistic strategies were identified, providing personalized treatment references for patients with advanced or drug-resistant breast cancer. Kun Wang and colleagues performed drug screening on 46 successfully established breast cancer organoid models, finding that 31 of them were highly sensitive [61]. The predictive accuracy of drug screening for clinical response was 78.4% (95% CI: 64.9%–91.9%). In 2024, Yuxin Cui et al. integrated breast cancer organoids with genomics and signaling pathway analyses to link mutations with drug responses [62]. On this basis, they combined organoids with a mini-PDX system to establish an *in vitro* rapid screening-*in vivo* validation platform. Breast cancer organoids can be used to systematically evaluate chemotherapeutic, targeted, and endocrine therapies, revealing differences in drug sensitivity among breast cancer subtypes across patients, and can recapitulate primary tumors at the levels of 3D structure, genetic characteristics, and function. In the same year, Xinxin Rao et al. established breast cancer organoids derived from patients with triple-negative breast cancer [63]. They confirmed that the histology and protein expression of these organoids were consistent with the original tumors. Based on this, a high-throughput drug screening platform was developed to screen epigenetic compounds, identifying agents targeting HDAC, JAK/STAT, histone demethylases, and Aurora kinases as having significant anti-tumor effects. A microarray chip-based drug screening platform was also developed using patient-derived breast cancer organoids, enabling low-volume, high-throughput evaluation of sensitivity to chemotherapeutic drugs and combination regimens [64]. Breast cancer organoid models can effectively distinguish drug response differences among organoids derived from different patients, thereby enabling the screening of potential drugs capable of reversing drug resistance.

Although traditional clinical treatment strategies have been effective, the persistently high mortality rate of breast cancer may be largely attributed to the drug resistance of cancer cells [65]. The establishment of breast cancer organoid models has introduced new technologies for tumor research, enabling more effective drug screening studies. Breast cancer comprises multiple subtypes, with approximately 70% being estrogen receptor-positive (ER+). However, about 30% of ER+ breast cancers are resistant to Tamoxifen. Therefore, breast cancer organoid models have been used to investigate mechanisms of drug resistance, and it has been found that bufalin can exert cytotoxic effects on Tamoxifen-resistant breast cancer cells [66]. In long-term drug screening experiments, traditional cell-based assays may develop a certain degree of drug resistance. Thus, using breast cancer organoid models for drug screening, combined with circulating tumor DNA (ctDNA), allows for the evaluation of cellular sensitivity and resistance mechanisms [67]. Breast cancer organoid models established from 25 patient samples were used to screen 13 clinically relevant drugs. The results confirmed significant interpatient variability in drug responses. These models can provide a comprehensive and near-real-time representation of tumor evolution, enabling early identification of drug resistance, real-time assessment of therapeutic efficacy, and precise adjustment of treatment strategies for personalized therapy.

Breast cancer organoids can effectively recapitulate the drug responses of primary tumors. They simulate the drug resistance process of cancer cells, evaluate combination therapy strategies, and guide re-treatment regimens for breast cancer patients with recurrence and metastasis. Integrating high-throughput drug screening platforms with organoids enables more efficient identification of mechanisms of cancer drug resistance.

#### 2.3.4 Predictive Therapeutic Drugs

Studies on breast cancer have shown that it is a highly heterogeneous cancer, and interpatient tumor heterogeneity leads to differences in therapeutic drug regimens used for tumor tissues. Therefore, it is necessary to establish breast cancer organoid models to study and predict treatment strategies for drug response (Fig. 2).

Traditional cell culture methods may lead to the loss of certain features of the original tumor tissue, resulting in inaccurate experimental outcomes. Therefore, breast cancer organoid models are required to accurately predict tumor drug sensitivity. Using patient-derived breast cancer organoid models, it has been validated that oxypalmatine (OPT) inhibits the growth of breast cancer organoids in a dose-dependent manner. OPT is a novel natural anti-breast cancer candidate drug that inhibits the PI3K/AKT signaling pathway, suppresses proliferation, and induces apoptosis [68]. In TNBC organoid models, the efficacy of IN10018 and crizotinib was studied, showing that inhibition of tumor cell proliferation is associated with targeting the cell cycle and apoptosis [69]. The tumor suppressor gene p53 is a key regulator of apoptosis, and combined treatment with IN10018 and crizotinib can upregulate p53 expression, thereby inhibiting cancer cell proliferation. Accurately predicting the drug sensitivity of cancer cells is an effective approach for TNBC treatment research. By establishing breast cancer organoids, models that are relevant to the clinic can be developed to predict drug sensitivity. As effective experimental tools, they overcome limitations of traditional models, such as the inability to faithfully simulate the complexity of tumor tissues. Docetaxel and carboplatin act on HER2-positive breast cancer organoids, while the combination of docetaxel and epirubicin shows synergistic effects in HER2-negative organoids, enabling the study of cancer cell sensitivity and drug resistance [70].

Breast cancer organoids can effectively preserve the molecular characteristics and heterogeneity of primary and metastatic tumors, and are therefore widely used for *in vitro* drug sensitivity testing and treatment response prediction. Using breast cancer organoid models for predictive drug screening allows detection of cellular sensitivity and drug resistance that may not be captured by certain gene-based testing methods. These models are also superior to prediction approaches that rely solely on receptor status or genetic mutations [71]. Breast cancer organoid models can be used to predict the efficacy of chemotherapeutic drugs, targeted therapies, and certain novel combination regimens. The screening results are often consistent with clinical treatment outcomes. Because breast cancer organoids preserve genomic alterations, phenotypic features, and tumor heterogeneity, they are used to predict drug sensitivity and cellular resistance [72]. Before clinical treatment, breast cancer organoid models can be used to evaluate whether HER2-overexpressing breast cancer patients are sensitive to HER2-targeted therapies, thereby enabling more precise therapeutic decisions [73]. Organoid models can accurately simulate the genetic alterations, phenotypic characteristics, and biological behaviors of patient tumors *in vitro*. Researchers can test the sensitivity of a large number of drugs on patient-derived organoids within a short period, screen for the most promising candidates, and further evaluate the efficacy of combination therapies.

For the treatment of breast cancer, there are various clinical therapeutic approaches, including surgery, radiotherapy, chemotherapy, and immunotherapy. However, since these treatment strategies are influenced by the heterogeneity among different breast cancer patients, there are significant differences in therapeutic outcomes. Precision treatment for breast cancer is a newly proposed therapeutic approach in which patient-derived breast cancer tumor tissues are directly treated with drugs, generating samples with altered epigenetic states and/or signaling pathways [74]. Based on these data, the therapeutic effects in different patients can be predicted, thereby allowing the selection of the most effective treatment strategy.

Studies based on patient-derived breast cancer organoids, tumor tissue fragments, and *in vivo* models have shown that precision therapeutic strategies designed according to multiple co-existing molecular alterations can help improve patient treatment outcomes [75]. By integrating bulk sequencing and single-cell analysis of primary tumors in breast cancer organoid models, tumor intratumoral heterogeneity and its evolutionary process have been systematically elucidated [76]. On this basis, therapeutic targets and intervention strategies with potential for precision medicine have been identified. Breast cancer organoids can recapitulate tumor genetic features, molecular markers, and cellular architecture, while genomic analysis helps identify potential therapeutic targets. Therefore, Vahid Niazi et al. integrated organoid models with genomic analysis [77]. As an important model for precision therapy, breast cancer organoids can reflect genetic mutations in patient tumor tissues. By analyzing the gene profiles of organoids, mutations can be identified and targeted therapeutic strategies can be developed. Using patient-derived breast cancer organoid models to study *BRCA1/2* mutations, it was found that targeted therapy with PARP inhibitors can improve clinical outcomes [78]. However, during the proliferation of breast cancer organoids, certain cancer cells may recruit invasive stromal components, leading to slowed organoid growth [79]. In addition, drug responses are predicted in breast cancer organoid systems lacking immune cells, which reduces the accuracy of experimental results, and these organoid models cannot fully recapitulate the tumor microenvironment.

Breast cancer organoids, as an effective tool for predicting therapeutic drugs, can predict drug sensitivity in advance before clinical treatment, thereby reducing ineffective therapies and avoiding toxic side effects. They, together with emerging technologies, are used to study breast cancer and to predict individualized treatment responses.

#### 2.3.5 Co-Culture of Breast Cancer Organoids with Immune Cells

Although breast cancer organoid models have outstanding advantages and promising potential in preclinical research, the translational application of organoid-based precision cancer therapy to clinical treatment remains elusive. For example, as mentioned earlier, breast cancer organoids lack immune cells and vascular networks within the microenvironment, and this limitation may affect their ability to simulate drug responses in patients *in vivo* (Fig. 2).

Therefore, combining immune cells with organoid models is more conducive to the clinical application of chemotherapy, targeted therapy, and immunotherapy. Diana P. Saraiva and colleagues successfully established a co-culture model of triple-negative breast cancer organoids and immune cells in 2020 [80]. Immune cells were able to survive in the 3D culture system and establish stable cell-cell interactions with organoids, thereby simulating the *in vivo* tumor immune microenvironment. This co-culture model significantly affected the biological behavior of tumor cells and the expression of immune-related factors. If experimental models lack the potential for immune editing, the real-time dynamics of clonal evolution dynamics may be affected [81]. Tumor-associated macrophages (TAMs) and breast cancer cells were co-cultured in a specific alginate cryogel. Because the gel can precisely replicate the physical and mechanical properties of the ECM, it enhances cancer invasion phenotypes such as growth, proliferation, migration, and ECM remodeling in breast cancer organoids [82]. On this basis, it is more effective to study invasive phenotypes, cytokine secretion, gene expression, and protein interactions during breast cancer progression. However, co-culturing only TAMs in alginate cryogel lacks fibroblasts and vascular endothelial cells, which may lead to incomplete reconstruction of the immune microenvironment. Immune checkpoint inhibitors (ICIs) enhance the immune system’s ability to recognize and attack tumor cells, and when combined with metabolic reprogramming, they can target the tumor microenvironment [83]. If ICIs combined with metabolic reprogramming are applied to breast cancer organoid models, the activity of immune cells within organoids may be enhanced. Future studies on breast cancer organoids may attempt to integrate these approaches to better explore the *in vitro* tumor microenvironment. In addition, immune cells can migrate toward and infiltrate breast cancer organoids in a 3D system. In a 3D co-culture of breast cancer organoids with primary macrophages, macrophages can actively migrate into and invade organoid structures, significantly altering their function and phenotype, causing organoids to exhibit TAM-like characteristics. This increases molecular heterogeneity and lipid complexity, successfully recapitulating key models such as paclitaxel resistance [84]. Establishing drug-resistant breast cancer organoid models allows the study of patients who do not respond to paclitaxel, thereby enabling the use of effective therapeutic drugs in clinical practice. Breast cancer organoid-immune cell co-culture systems can generally be divided into three types: autologous immune cell models, stromal-associated immune models, and immune function-oriented models [85]. These three co-culture models compensate for the lack of immune components in traditional organoid systems when simulating the tumor microenvironment *in vitro*. Breast cancer organoid-immune co-culture models have been applied to analyze mechanisms of immune regulation, tumor immune evasion, and differences in immunotherapy responses among breast cancer subtypes. When simulating the tumor microenvironment using breast cancer organoids, T cell activation plays a major role in immune responses [86]. Peripheral blood mononuclear cells (PBMCs) derived from breast cancer patients were co-cultured with patient-derived breast cancer organoids for two weeks to verify whether human T cells in organoids respond to the HEKLA-Exos vaccine [87]. When organoids were treated with HEKLA-Exos, their growth was significantly inhibited and cell death was induced. HEKLA-Exos selectively induces immunogenic cell death (ICD) in breast cancer cells, thereby promoting the growth of type I conventional dendritic cells (cDC1) and activating CD8^+^ T cells *in vitro*. cDC1 serves as a bridge between innate and adaptive immunity by transmitting information from breast cancer organoid tumor cells to CD8^+^ T cells. CD8^+^ T cells then recognize and attack breast cancer cells, thereby achieving therapeutic effects.

Breast cancer organoid-immune cell co-culture models provide a more realistic platform for studying interactions between tumor cells and immune cells by better simulating the tumor immune microenvironment.

#### 2.3.6 Establishment of Vascularized Breast Cancer Organoids

The physiological function and biological activity of regenerated tissues depend on vascularization, which ensures a continuous supply of oxygen and nutrients [88]. It also facilitates the delivery of therapeutic drugs into tumor tissues and enables the removal of metabolic waste [89]. If breast cancer organoids lack a functional vascular system, it may lead to increased metabolic demand, resulting in necrosis in the core region. Co-culturing organoids with microvessels or endothelial cells can enable the formation of vascular networks within organoids. Vascularized breast cancer organoids promote endothelial sprouting and the formation of secondary vascular branches. The ECM regulates vascularized breast cancer organoids, promoting vascularization and optimizing tissue morphogenesis [90]. When organoids are used to simulate the *in vivo* microenvironment, capillaries provide essential nutrients for cancer cell development and also serve as carriers for anti-tumor drug delivery. Insufficient vascular growth becomes a limiting factor for subsequent dynamic monitoring of cancer cell intravasation [91]. Vascularized breast cancer organoids can more accurately simulate tissue microphysiological functions and reconstruct pathological features of human diseases *in vitro* [92]. Breast cancer organoids with vascular networks constructed on a PDMS microfluidic platform can be functionalized by using human umbilical vein endothelial cells on the inner surface of the lumen, thereby mimicking the physiological structure and barrier function of natural blood vessels [93]. This barrier can regulate the transport of oxygen, nutrients, metabolic waste, and drugs between blood and tumor tissues, enabling better simulation of the *in vitro* tumor microenvironment. A 3D hydrogel matrix containing breast cancer organoids was co-cultured with a human microvascular network on a microfluidic chip. Perfused microvascularized breast cancer organoid models showed that both vascular hyaluronic acid depletion and vascular barrier dysfunction were induced by interleukin-8 (IL-8) [94]. Researchers used vascularized breast cancer organoids to more accurately analyze the causes of drug delivery barriers and to test potential standardized strategies for drug delivery to tumor cells. Administration of doxorubicin in vascularized breast cancer organoids revealed the drug’s vascular permeability and the physical characteristics of its transendothelial transport into surrounding stroma [95]. This helps optimize drug distribution and penetration across vascular barriers toward tumor cells, providing a clearer understanding of drug delivery mechanisms and improving drug screening. A breast cancer organoid model was established using the ductal breast cancer cell line ZR-75-1, which maintained epithelial characteristics and high proliferative activity in Matrigel 3D culture. By combining breast cancer organoids with a vascularized bioengineering system (sVEB), a vascularized breast cancer model was constructed [96]. This model recapitulates the interactions between cancer cells and blood vessels within the tumor microenvironment. Kyle H. Vining found that stem cells are regulated by mechanical properties and forces, which can guide developmental processes and tissue repair. Studying mechanical gradients in breast cancer organoids may better simulate the human microenvironment.

Vascularized breast cancer organoids can recapitulate the tumor microenvironment and individual heterogeneity. As a model used to evaluate drug efficacy and develop personalized precision therapies, they help identify populations with high therapeutic benefit and improve the precision of clinical drug use [97].

## 3 The Challenges of Breast Cancer Organoids

Breast cancer organoids can better preserve the genetic and phenotypic characteristics of primary tumors in *in vitro* tumor response assays and are currently an ideal model for studying tumor tissues. However, successfully establishing organoid models from patient tumor tissues remains a challenge for many laboratories. Norman Sachs et al. successfully cultured 95 organoid models from 155 patient samples. The success rate of colorectal cancer organoid establishment was 80.21%, with 77 organoid models successfully generated from 96 patient samples [98]. The relatively high survival rate may be attributed to strict control of processing time during sample handling, ensuring that the interval between surgical resection and *in vitro* culture of colorectal cancer tissues did not exceed 30 min. Among 72 pancreatic cancer patient samples, including 51 from treatment-naive patients and 21 from patients receiving chemotherapy, the success rates of pancreatic cancer organoid establishment were 76% and 71%, respectively [99]. The high success rates of colorectal and pancreatic cancer organoids may be due to the preferential use of treatment-naive patient samples in the studies, thereby improving model establishment efficiency. In lung cancer organoid construction, Han-Min Wang et al. successfully established 162 lung cancer organoids from 214 patient samples, with a success rate of 75.7% [100]. Their study indicated that samples obtained by needle biopsy contain a limited number of cells, making organoid establishment more difficult. Improving the success rate of breast cancer organoid establishment relies on integrating and optimizing the established experience from the above three types of organoid systems.

**Figure 3:**
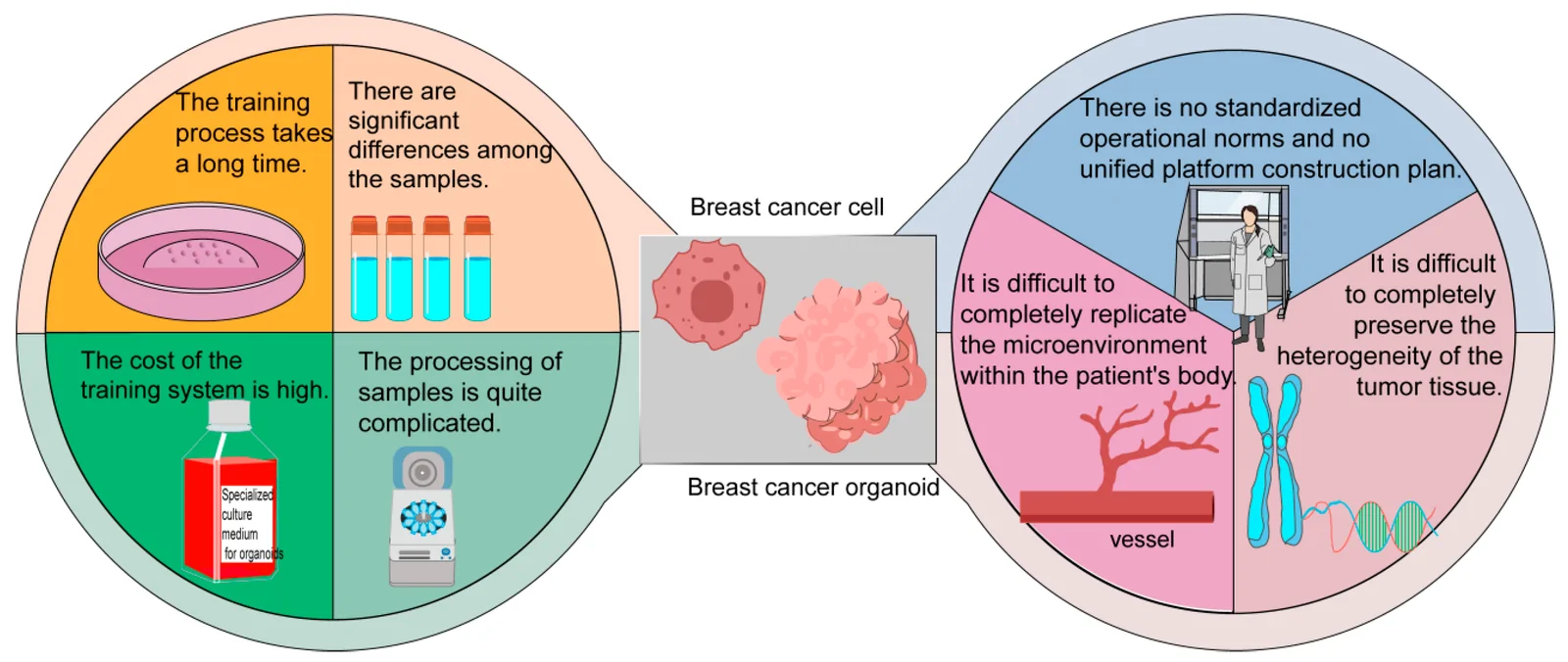
**Challenges and limitations of breast cancer organoids.** Breast cancer organoids have several limitations. For example, the culture period is long, as it takes about 28 days to generate organoids from breast cancer tissue samples. There are significant differences in the microenvironment among breast cancer organoid models. The cost of culture is high, requiring expensive growth factors and matrix gels. The procedures are complex, with a lack of standardized protocols and unified construction platforms; different laboratories use different methods as well as different combinations of inhibitors and growth factors when culturing breast cancer organoids. Breast cancer organoids also lack a complete vascular network and are difficult to fully recapitulate the *in vivo* microenvironment of patients. In addition, they are unable to completely preserve the heterogeneity of tumor tissues. (Using Adobe Illustrator 2024 to create images).

While focusing on improving success rates, the impact of differences in patient sample sources on culture outcomes cannot be ignored. Tumor tissues from different patients show significant differences in the microenvironment and other features even in successfully established breast cancer organoid models (Fig. 3). Even when breast cancer samples from the same source are cultured under identical conditions, it is still difficult to stably obtain organoids with highly consistent phenotypes [101]. The reason for this phenomenon may be that the cultivation of breast cancer organoid models has not yet formed standardized operating protocols and a unified platform construction scheme. Different laboratories use different methods, as well as different combinations of inhibitors and growth factors, for breast cancer organoid culture. In addition, with the increasing complexity of the tumor microenvironment in breast cancer tissues, the culture period has also correspondingly increased. The median time required from breast tumor tissue to the establishment of breast cancer organoids is 28 days (Table 1) [102]. To address these limitations, researchers have combined the agile reconfigurable segmented organoid (SOAR) bioprinting technology with patient-derived ECM proteins to more precisely mimic the tumor microenvironment [103]. SOAR bioprinting technology can culture biopsy samples into breast cancer organoid models within 7 days, and the resulting patient-derived organoids show good reproducibility.

**Table 1:** Comprehensive Indicators and Key Parameters of Breast Cancer Organoids.

Comprehensive Evaluation Index	Key Parameters
The scale of the breast cancer organoid biological sample library	>100 cases
Constructing a cycle of breast cancer organoids from patient sources	Median time: 28 days
Cost of constructing patient-derived breast cancer organoids	The main costs come from the expensive growth factors and matrix gel
The consistency rate between drug predictions for breast cancer organoids and clinical outcomes	78.4%

Breast cancer organoid models provide an important technological breakthrough for tumor biology, establishing three-dimensional models that are closer to the complex *in vivo* microenvironment of patients than traditional culture systems. They recapitulate the structural characteristics of tumors and serve as powerful tools for elucidating the key mechanisms underlying the initiation and progression of breast cancer, thereby expanding research pathways for novel therapeutic strategies. Future challenges for breast cancer organoids remain in integrating components of the microenvironment to increase the complexity of *in vitro* systems.

## 4 The Opportunities of Breast Cancer Organoids

Studies have indicated that the maturity of the culture system has a greater impact on organoid establishment success than the intrinsic characteristics of the tumor itself [104]. When breast cancer organoids are embedded and cultured in Matrigel, the size and composition of the organoids often exhibit high variability. This phenomenon leads to significant heterogeneity among different organoids. In addition, the composition of the matrix gel required for organoid culture is not clearly defined, and it varies between batches, resulting in poor reproducibility of experimental results and thus limiting its application in large-scale drug screening [105]. If the protein composition of the matrix gel is adjusted, for example by dilution or by adding collagen, the reproducibility of organoid culture can be improved. Madeline A. Lancaster et al. did not use patterning growth factors when culturing cerebral organoids, yet successfully induced organoids to better exhibit the intrinsic mechanisms of the human brain [106]. This approach may also be attempted in the culture of breast cancer organoids.

After the successful establishment and culture of breast cancer organoids, they are applied to high-throughput drug screening. The predictive sensitivity of breast cancer organoids for drug response is 84.8%, whereas that of colorectal cancer organoids is 63.33%. This indicates that breast cancer organoids have achieved a relatively high level of technical maturity in evaluating drug sensitivity. However, the specificity of drug screening in pancreatic cancer organoids reaches 92.9%, while that of breast cancer organoids is only 25.0%, suggesting that current models still have limitations in excluding clinically ineffective drugs [107]. Combining breast cancer organoids with leaf-vein-inspired microfluidic chips can improve the specificity of the organoid model [108]. These microfluidic chips, by constructing a biomimetic fluidic environment, can simulate the patient’s vascular system, thereby accurately recapitulating complex *in vivo* cellular signaling pathways and enabling more realistic prediction of drug-specific efficacy *in vitro* [109].

In preclinical cancer research prior to clinical treatment, how to establish models that recapitulate the complex *in vivo* microenvironment of patients and fully preserve tumor heterogeneity remains a key issue that organoid models need to address. Due to the immaturity of culture systems, breast cancer organoids typically lack critical regulatory components of the tumor microenvironment, mainly including immune cells, cancer-associated fibroblasts, and vascular networks [110]. This discrepancy may lead to differences between drug responses observed in experiments and clinical outcomes after treatment [111,112].

Overall, the core development of the breast cancer organoid field will focus on continuously optimizing culture systems to further improve the pathological relevance and genetic stability of organoids. Breast cancer organoid models enhance the ability to simulate tumor heterogeneity and the microenvironment, promoting their advancement toward more advanced mechanistic studies and clinical translational applications.

## 5 Conclusion

Breast cancer organoid models are a highly promising preclinical research platform with significant value in cancer studies. They can simulate the key processes of breast cancer initiation and progression *in vitro* in a patient-specific manner. Combined with genetic manipulation approaches, organoid models can elucidate tumor genes and regulatory networks, serving as an effective tool for high-throughput drug screening and precision medicine [113].

Breast cancer organoid models are successfully established from surgical or biopsy specimens. However, due to limitations such as high construction difficulty, high cost, and long culture periods, further breakthroughs are needed for their long-term stable expansion [114]. Breast cancer organoid models can simulate the tumor microenvironment *in vitro*, exhibiting good stability, low mutation rates, and the ability to effectively recapitulate the tissue specificity of patient tumors. They better model cell-cell interactions, as well as tissue structure and functional characteristics, making them more consistent with physiological conditions in the human body [115]. Breast cancer organoid models can accurately simulate tumor tissue characteristics and have become important models for studying tumor initiation, invasion and metastasis, drug responses, and toxicity evaluation. They also enable the analysis of cancer stem cell functions and, through exploring organoid-based target discovery, promote the development of more precise and less toxic therapeutic strategies.

Breast cancer organoid models have been used to simulate immunotherapy responses, accelerating the preclinical evaluation of related therapeutic strategies [116]. An increase in gene expression levels of immune response pathway genes is a strong predictor of treatment outcomes [117]. By co-culturing immune cells, stromal cells, and integrating microfluidic chip technologies with breast cancer organoids, the tumor microenvironment can be more realistically recapitulated. Breast cancer organoids enable accurate assessment of the potential of immune checkpoint inhibitors, CAR-T/NK cell therapies, and combination therapies, providing screening strategies for personalized drug sensitivity testing and precision treatment [118]. Breast cancer stem cells isolated from patient tumor tissues can be cultured into organoid models *in vitro*, preserving key tumor characteristics and being used to systematically study the initiation and progression of breast cancer. Therefore, they demonstrate important application value in patient-specific disease modeling, high-throughput anti-tumor drug screening, and tumor microenvironment engineering [119].

Clinical decision support systems (CDSS) combined with explainable artificial intelligence (XAI) can be used to predict treatment responses and survival outcomes in malignant tumors [120]. If XAI-CDSS is integrated with breast cancer organoids, the effectiveness of organoid-based drug screening is further enhanced, accelerating the translation from laboratory research to clinical application. With the continuous integration of biomaterials and engineering technologies into organoid culture systems, the ability of breast cancer organoid models to mimic *in vivo* environments has been significantly improved, with ongoing enhancements in stability, reproducibility, and application breadth [121]. Vascularized breast cancer organoids constructed using microfluidic chips recreate the tumor tissue microenvironment and deeply simulate *in vivo* metabolism, microenvironmental conditions, and cell-cell interactions, representing a major breakthrough in physiologically relevant 3D modeling and precise disease simulation. Compared with traditional breast cancer organoid models, immune cell co-culture organoid systems can more accurately simulate the tumor microenvironment *in vitro* and are closer to the physiological state of patients. Traditional breast cancer organoid culture conditions are usually static, whereas microfluidic-chip-based organoid systems can mimic the dynamic perfusion characteristics of blood vessels *in vivo*, thereby facilitating the study of drug action mechanisms. The combination of immune cells and microfluidic technologies in breast cancer organoid co-culture systems may further increase the complexity of the *in vitro* tumor microenvironment. The integration of breast cancer organoid models with cutting-edge technologies such as synthetic biology and gene editing marks a transformative era in biomedical research. This synergistic model provides opportunities to develop innovative therapeutic strategies, optimize drug screening, and advance research on complex diseases. With the continuous development of breast cancer organoid technology, the link between laboratory research and clinical application has become increasingly solid, laying a strong foundation for the advancement of precision medicine.

3D organoid culture models provide a new platform for clinical diagnosis, treatment, and cancer research. Breast cancer organoid models are established using tissue samples directly derived from patients to simulate the biological characteristics of breast cancer. This largely recapitulates the histological structure and genomic features of the original breast cancer tissue and can simulate the heterogeneity of the primary tumor *in vitro*. Breast cancer organoids provide an important research platform for modeling individual patients’ tumors, studying disease mechanisms, predicting drug responses, and developing personalized clinical treatment strategies. Breast cancer organoid models are at the forefront of biomedical development, with broad and far-reaching potential impact. With continuous exploration by researchers and clinicians, this model has the potential to facilitate the development of innovative therapeutic strategies. The establishment of mature breast cancer organoid models and the advancement of precision personalized therapy will become the core direction of future organoid research.

## Data Availability

Not applicable.
